# Misdelivery at the Nuclear Pore Complex—Stopping a Virus Dead in Its Tracks

**DOI:** 10.3390/cells4030277

**Published:** 2015-07-28

**Authors:** Justin W. Flatt, Urs F. Greber

**Affiliations:** Institute of Molecular Life Sciences, University of Zürich, Winterthurerstrasse 190, 8057 Zürich, Switzerland

**Keywords:** nuclear import/transport, gene therapy, Nup, importin/karyopherin, click chemistry, virus entry, uncoating/disassembly, innate immunity, interferon, MxB

## Abstract

Many viruses deliver their genomes into the host cell’s nucleus before they replicate. While onco-retroviruses and papillomaviruses tether their genomes to host chromatin upon mitotic breakdown of the nuclear envelope, lentiviruses, such as human immunodeficiency virus, adenoviruses, herpesviruses, parvoviruses, influenza viruses, hepatitis B virus, polyomaviruses, and baculoviruses deliver their genomes into the nucleus of post-mitotic cells. This poses the significant challenge of slipping a DNA or RNA genome past the nuclear pore complex (NPC) embedded in the nuclear envelope. Quantitative fluorescence imaging is shedding new light on this process, with recent data implicating misdelivery of viral genomes at nuclear pores as a bottleneck to virus replication. Here, we infer NPC functions for nuclear import of viral genomes from cell biology experiments and explore potential causes of misdelivery, including improper virus docking at NPCs, incomplete translocation, virus-induced stress and innate immunity reactions. We conclude by discussing consequences of viral genome misdelivery for viruses and host cells, and lay out future questions to enhance our understanding of this phenomenon. Further studies into viral genome misdelivery may reveal unexpected aspects about NPC structure and function, as well as aid in developing strategies for controlling viral infections to improve human health.

## 1. Introduction

Viruses are highly efficient gene delivery devices when compared to alternative approaches such as naked nucleic acid delivery or artificial vectors, such as polyamine or liposome complexes [1,2,3]. Viral vectors are actively harnessed in the clinics and model organisms to deliver genes to diseased cells and tissues where they can complement deficiencies or mitigate uncontrolled cell growth in cancer [4,5,6,7]. Yet, it becomes increasingly clear that innate immunity and host defense restrict viral infections not just during replication and virus production, but also during entry [8,9,10,11]. In the case of DNA viruses and lentiviruses, immunity is frequently directed against the viral DNA (vDNA) in endosomes, the cytosol, and the nucleus [12,13,14,15,16]. This can give rise to inflammasome or interferon activation, which is capable of restricting both the infection and transduction efficacy of viral vectors. 

Viruses that replicate in the intact cell nucleus must transport their genomes through the nuclear pore complex (NPC) embedded in the nuclear envelope [17,18,19,20,21,22,23,24]. By contrast, onco-retroviruses and papillomaviruses wait for the nuclear envelope to break down in mitosis, whereafter they tether to host chromatin and are planted in the reforming nucleus at telophase. In both cases, nuclear access requires that the viral genome be freed from the enclosing capsid. 

The separation of the viral genome from the capsid is termed uncoating. This critical reaction exposes the genetic information and is key to making progeny virions. It is a stepwise gain of function process, which starts at the cell surface upon virus docking to receptors [25] and involves protein and lipid signaling [26,27]. The large majority of viruses penetrate host cell membranes via endosomes [12,28,29,30] and connect to cytoplasmic transport machineries for subsequent acto-myosin or microtubule-based transport [31,32,33,34].

Most viruses that replicate in the nucleus are larger than the translocation channel of the NPC, which can accommodate cargo with a maximal size of about 40 nm [35]. Since the genome is smaller and more flexible than the capsid, uncoating likely occurs before the genome is translocated into the nucleus [36,37,38]. Similar to the host’s cellular cargo, viruses use nuclear localization sequences (NLSs), soluble karyopherins of the importin-beta (Imp-b) family, such as Imp-a, Imp-b or transportin, as well as Ran-GTP to transport their genetic cargo through the nuclear pore complex (NPC) and into the nucleus. Given the large diversity of viral agents replicating in the nucleus, it is not surprising that there are multiple strategies to get past the NPC. Recent data indicate that viral genome transport through the NPC is not always smooth sailing, but can be blocked or aborted. This may be a basis for host defense mechanisms that evoke an intrinsic antiviral response. 

## 2. The NPC — A Restriction Point in Cellular Trafficking

The nuclear pore complex (NPC) serves as both gate and gatekeeper to the nucleus. The NPC is a disk-like structure about 50 × 100 nm in size. NPCs are assembled from multiple copies of around 30 different proteins, called nucleoporins (Nups) [39,40,41,42]. Peripheral Nups, such as Nup358 and Nup214, which are located at the cytoplasmic face of the NPC, possess a high degree of structural flexibility, whereas the scaffold Nups adopt a more stable configuration that is important for anchoring the NPC in the nuclear membrane [43]. 

NPCs control the passage of a wide range of cargos through passive diffusion and facilitated translocation [44,45,46]. Passive diffusion is possible without the input of chemical energy or soluble factors for cargo smaller than 2–3 nm (equivalent to a globular protein of ~40 kDa). Each pore, which spans the outer and inner nuclear membranes, is capable of handling approximately 1000 translocation events per second, corresponding to a mass flow of nearly 100 MDa/s [47]. Several models have been proposed to explain how NPCs achieve such fast and selective transport while at the same time avoiding traffic jams. Transport models include the virtual gating model [48], the selective phase model involving phenylalanine-glycine (FG)-repeat hydrogel forming Nups [47], and the entropic barrier model [49]. 

How the NPC achieves size-selective translocation of cargo across the nuclear envelope has been one of the most intriguing unsolved problems in cellular trafficking. Single-molecule methods are starting to provide details on how translocation works for large substrates on the level of ribosomal subunits and viral cargoes [50,51,52]. For example, a recent report used a quantum dot-based cargo, which was covalently linked with multiple copies of the importin beta binding (IBB) domain of snurportin-1 to monitor import. The IBB-linked quantum dot cargo docked on the cytoplasmic NPC filaments for a few milliseconds, after which only a small fraction entered the central channel [52]. Remarkably, this initial step was shown to be rather inefficient as 75% of the events were rejected to the cytoplasm. Of the remaining cargo about 80% passed beyond the size-selective gate, and migrated within the central transporter region by anomalous subdiffusion along the transverse and transport axes. The irreversible exit step at the nuclear basket mediated by Ran-GTP was shown to have an efficiency of approximately 50%, yielding an estimated overall import efficiency of 10%. Such work increases our understanding of how single large cargos translocate through nuclear pores but may not accurately reflect the transport efficiency for smaller substrates.

## 3. The NPC — A Bottleneck in Virus Entry?

**Figure 1:**
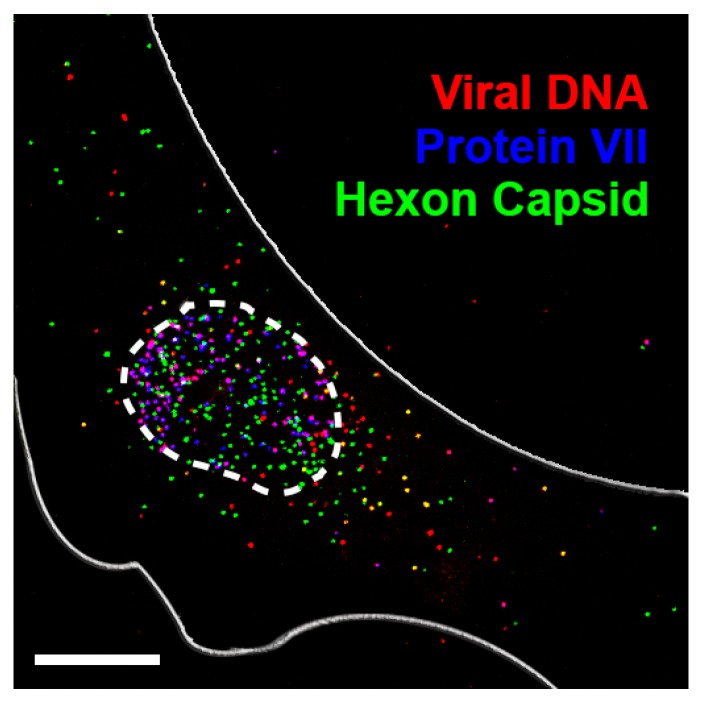
Misdelivery of capsid-free adenovirus DNA after uncoating at the nuclear pore complex. Wang *et al.* [53] infected HeLa cells for 150 minutes followed by fixation and viral genome click-labeling using Alexa Fluor 594 (red). Shown is a total projection of confocal optical slices depicting immune-stained protein VII (blue) and hexon capsid (green). Viral genomes associated with protein VII appear as purple dots in the nucleus, whereas partially uncoated cytosolic virus particles containing viral DNA (vDNA) appear as yellow dots. It is thought that protein VII remains attached to incoming vDNA during translocation into the nucleus, but copy number per genome is uncertain, as well as if any other viral proteins, such as terminal protein or protein X remain associated during import [58,59,60]. Analyses revealed that a large pool of viral DNA does not reach the nucleus during infection, but rather accumulates capsid-free in the cytosol of the host cell. The borders (white) were identified using DAPI for the nucleus and contrast-enhanced protein VII for the cell membrane. Scale bar, 10 µm.

Recent single-genome tracking experiments have provided a fresh perspective on viral nuclear import by permitting a first glimpse of misdelivery at the NPC [53]. It was demonstrated specifically for human adenovirus (HAdV) that a significant fraction of incoming viral genomes do not make it into the nuclear compartment for replication upon virus docking and uncoating at the NPC. Instead they accumulate as capsid-free vDNA in the cytosol (see Figure 1, and [53]). The amassing of these misdelivered cytosolic vDNAs was quantified by fluorescence microscopy, and found to vary considerably from cell to cell, with a large fraction not reaching the nucleus [53]. In cells treated with leptomycin B, a drug that blocks HAdV particles from attaching to NPCs [54,55], misdelivery of capsid-free vDNA to the cytosol was completely abrogated [53]. This indicated that uncoating cues from the NPC are involved in vDNA misdelivery. 

The issue is of general interest as misdelivery may represent an ‘Achilles’ heel’ for viruses infecting non-dividing cells. For example, over half of human immunodeficiency virus-1 (HIV-1) reverse-transcribed genomes accumulate as capsid-free dead-end products in the cytosol of primary human macrophages [56,57]. Could it be that incoming HIV-1 genomes fail to transport to the nucleus upon docking at NPCs? Better understanding of how viral genome misdelivery occurs may help in designing strategies to keep viruses out of the nucleus, as well as suggest ways of improving therapeutic gene delivery efficiency. Below, we outline the possible ways that NPCs stop viruses dead in their tracks during cell entry and discuss how this may influence viral infections.

## 4. Why the Gates of the Nucleus May not always Open for Viruses

For viruses, residence inside cells is generally short-lived. The reasons may be several fold. Viruses are unusual structures that do not normally occur in cells, and are subject to antiviral immunity [9,13]. As described above, viruses package their genomes into protective protein containers called capsids, which are not built to last but rather to disassemble in a tightly regulated, multi-step process termed uncoating. Uncoating is essential for infection, and enabled by cell- and also virion-associated factors [36,37,38,61]. Capsid disassembly is considered complete when the viral genome is released for replication. This final step in entry is particularly difficult to negotiate when it involves traveling across the NPC. 

**Figure 2:**
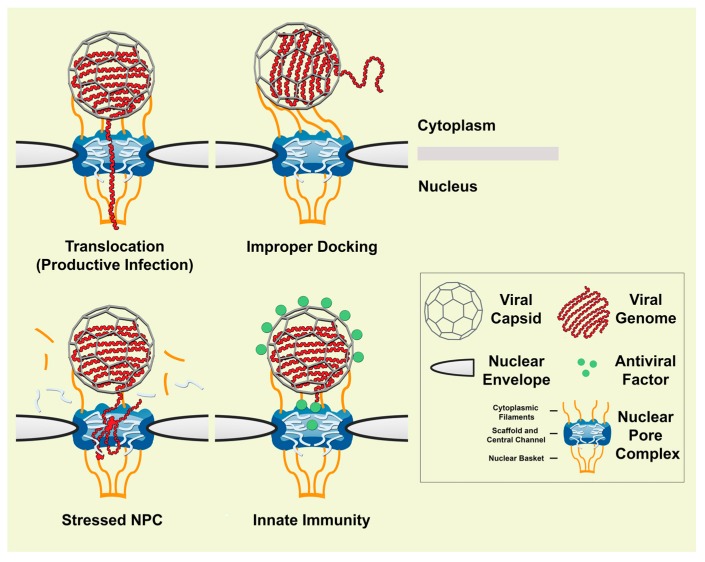
Possible ways that nuclear pore complexes inhibit viruses during cell entry. Viruses that replicate in the nuclear compartment of post-mitotic cells must somehow overcome the selective barrier of the nuclear pore complex (NPC). Large virus capsids, such as those of herpesviruses and adenoviruses are too big to pass directly through NPCs. These viruses have evolved mechanisms to negotiate translocation of their genomes and accessory proteins across NPCs (upper left). However, improper docking (upper right) and mechano-chemical stress (lower left) may cause incoming viral nucleic acids to be misdelivered to the cytosol rather than entering into the nucleus for replication. Likewise, antiviral factors, such as the recently reported interferon induced myxovirus resistance protein 2 (MxB) protein, may restrict uncoating and/or nuclear import of genomes from certain viruses, such as human immunodeficiency virus-1 (HIV) (lower right).

The NPC is a dynamic, selective, crowded environment that is constantly loaded with fast moving cargoes. In such an environment, bulky viral genomes push the limits of transport capacity and increase the probability of something going wrong. Probable causes of misdelivery include improper virus docking to NPCs, inefficient translocation of vDNA through NPCs, NPC stress, and innate immunity factors interfering with uncoating or translocation (Figure 2).

### 4.1. Improper Virus Docking to the NPC

The first critical step in viral nuclear import occurs when a virus or an uncoated subviral structure reaches a NPC and attaches itself to the surface. This process is governed by a series of intricate protein-protein interactions that are virus-specific, and can be divided into separate phases. Initially, viruses or subviral structures, such as DNA or RNA nucleoparticles target NPCs by association of viral NLS motifs with cellular transport receptors of the importin/karyopherin family [62,63,64,65,66,67,68,69,70,71,72,73]. Alternatively, certain viruses or viral proteins directly bind to Nups, such as Nup358 or Nup214 in the cytoplasmic periphery of the NPC [54,74,75,76,77,78,79]. 

Upon making contact, viruses position themselves correctly with respect to the cytoplasmic opening of the central channel of the NPC so that a released genome can pass through (Figure 2, upper left). This may be facilitated by the 8-fold radial symmetry of the NPC and the oligomeric nature of the virus capsid. Exactly how viruses and NPCs align is unclear, but may initially involve low affinity interactions with unstructured FG regions located within the highly mobile cytoplasmic filaments emanating from the NPC [80]. For example, HAdVs binding to the NPC can be intercepted by antibodies directed to the FG-region on Nup214, and the binding further depends on an N-terminal domain of Nup214 lacking FG-repeats [74,78]. Avidity increase resulting from multiple receptor binding sites on a virus likely guarantees nearly irreversible binding once the correct orientation has been achieved. 

That a virus is capable of docking to a heavily trafficked NPC for genome import is an impressive feat, and serves as a testament to both the structural ingenuity of viruses and the malleability of host cells. However, the process is far from perfect, as has been shown from studies of influenza A virus (IAV) nuclear import [81]. IAV has a single-stranded, negative-sense RNA genome that is packaged into eight unique rod-shaped segments called ribonucleoproteins (RNPs), each of which is associated with multiple nucleoprotein molecules and a viral polymerase [82]. Nuclear import of uncoated RNPs is mediated by the importin-α/β1-dependent nuclear import pathway [83]. Live-cell fluorescence imaging experiments have revealed that RNPs bind heterogeneously to NPCs with dissociation rates spanning two orders of magnitude, ranging from 1 to 100 seconds [81]. Such dissociation times imply that IAV stalls at the gate, especially considering that nuclear import of cellular cargoes normally only takes a few milliseconds [84,85,86]. An analysis of the import kinetics of IAV RNPs led to a model where RNPs undergo multiple rounds of binding and release before finally being translocated through NPCs [81]. This idea is intriguing given how little we currently know about the structure and dynamics at play when viruses attach and deliver genomes at NPCs. Clearly, it is necessary to further explore this aspect of viral nuclear import. It will be interesting to see, for example, whether improper docking (Figure 2, upper right) reduces the number of viral genomes entering the nucleus. 

### 4.2. Translocation of Viral Cargo

After a virus latches onto an NPC, the immediate challenge becomes delivering its genome and accessory proteins into the nucleus. Small viruses, such as parvovirus or hepatitis B virus capsids may enter the NPC largely intact, although little is known about the kinetics, efficiencies, and mechanisms [87,88,89]. Likewise, the brick-shaped baculovirus nucleocapsid has been observed in tight association with the NPC, suggesting considerable conformational flexibility of the NPC [90]. However, it remains unknown how efficient the translocation of baculovirus is. 

Large viruses comprise the vast majority of viruses replicating in the nucleus. These include adenovirus, herpesvirus, HIV, and influenza virus. For large viruses to access the limited space and enter the pore, partial or complete uncoating is required [54,61,77,91,92,93,94,95,96,97,98]. Uncoating releases a large amount of elastic energy as viral genomes are rapidly unpackaged and unfolded, giving rise to mechano-chemical stress [99,100,101]. Going into the pore, the situation worsens when an incoming large hydrophilic DNA genome complexed with viral proteins is forced to pass a tightly enclosed hydrophobic space against a steep concentration gradient composed of host chromatin. Any movement of large hydrophilic cargo is karyopherin/importin dependent, and may significantly widen the NPC central channel [102,103]. This might occur through the action of Imp-b, which is required for the translocation of vDNA in case of HAdV, HSV1 and HIV [68,69,74,104]. Imp-b mediates interactions with FG-domains of the NPC and is in a complex with Imp-a, which binds to cargo-NLS. Fluorescence recovery of photobleaching in combination with super-resolution fluorescence microscopy and RNA interference has recently shown that there is a stable pool of Imp-b at the NPC and that a fraction of this pool is turned over in a Ran-GTP dependent manner [45]. Remarkably, Imp-b but not transportin was found to alter the permeability of the NPC, and this was enhanced by Nup153, which binds Imp-b and transportin, but only Imp-b binding was abrogated by Ran-GTP. It is possible that if clogging, genome shearing, and misdelivery are avoided, the translocated viral genome emerges on the nuclear side of the pore, where it may be released in a manner requiring Imp-b, Ran-GTP and the basket protein Nup153. 

When nuclear import of adenoviral DNA was quantitatively assessed by click-chemistry labeling and fluorescence microscopy, it was found that only a fraction of the incoming viral genomes were imported into the nucleus [53]. At least three scenarios can be envisioned that explain this translocation inefficiency. In the first one, a significant fraction of viral genomes are displaced as a result of the kinesin-1 motor ripping the viral capsid apart (see section 4.3 below for details). This would lead to a direct misplacement of vDNA into the cytoplasm. A second scenario is that a subset of NPCs become damaged while under the stress of viral uncoating and/or translocation, such that the pathway into the nucleus is in need of repair. Under such conditions, viral genomes may enter pores, but only to a limited extent before returning to the cytoplasm (Figure 2, lower left). The third possibility is that vDNA misdelivery is a consequence of how the NPC normally functions, namely as a gate that rejects a large fraction of incoming cargo before entering the central translocation channel [52]. These scenarios pose exciting challenges and avenues for future research. 

### 4.3. Stressed NPC

In recent years, it has been discovered that the stress caused by virus uncoating can lead to a partial breakage of the NPC. Such was shown for HAdV, which is among the largest (~920 Å diameter) and most complex (~150 MDa) of the non-enveloped double-stranded DNA viruses [54,105,106]. A partly dismantled HAdV docks to the NPC through its major capsid protein hexon binding to Nup214, and uses one of its minor capsid proteins, pIX, to recruit the kinesin-1 motor, which is docked at Nup358. At this stage the genome is still fully enclosed [53]. The binding of kinesin-1 to Nup358 relieves auto-inhibition of the motor and allows kinesin-1 to translocate on microtubule (MT) filaments that are in close proximity of the NPC by virtue of the MT binding domain of Nup358 [107,108]. These data suggest that kinesin-1 exerts a pulling force against the NPC-docked HAdV, and thereby disrupts the capsid and sets free the viral genome. In support of this model, kinesin motors, capsid fragments and various Nups are observed to be displaced from the NPCs, as indicated by live cell fluorescence microscopy and fluorescence *in situ* photo-conversion experiments [54]. In addition to disrupting the viral capsid, kinesin-1 activity disrupts the integrity and functionality of the NPC by displacing Nup214, Nup358 and Nup62 into the surrounding cytoplasm. Nup62 is part of the central channel and together with other Nups, such as Nup96/98, is responsible for maintaining the selectivity barrier of the NPC [109]. Interestingly, Nup153 is not displaced from the NPC, suggesting that it has a function in translocation of the vDNA through the stressed NPC [54]. The vDNA imported into the nucleus is a linear double stranded molecule covalently attached to the terminal protein and condensed with the viral protein VII, and possibly the small protein X but devoid of the other DNA-associated protein V [58,59,110]. 

### 4.4. Innate Immunity at the NPC

Innate immunity is a collection of generic and immediate cellular defense reactions against pathogens without long lasting effects. It is a multi-branched system that acts as a physical and chemical barrier against intruding pathogens, and involves chemicals that recruit immune cells to the site of infection [111]. It comprises immune cell activation and clearance mechanisms of infected cells, and eventually leads to the activation of an adaptive immune response. Innate pathogen detection involves compartment-specific sensory receptors called pattern recognition receptors (PRRs) that recognize pathogen associated molecular patterns (PAMPs), such as microbial and viral lipids, proteins, nucleic acids, and sugars [112,113,114]. Recognizing these viral PAMPs at the different stages of virus entry modulates complex signaling pathways, which in turn induce distinct sets of effector mechanisms that block virus replication and promote viral clearance [115]. In addition, cell-autonomous immunity relies on danger receptors to detect danger associated molecular patterns (DAMPs) — namely, disturbances in homeostasis generated by the process of infection rather than the pathogen itself [116]. Such a built-in, multifaceted defense program, where antiviral factors tailored to specific cellular microenvironments target and suppress the different steps of the viral life cycle, greatly reduces the probability of a productive infection. 

To date, several classes of membrane bound and cytosolic receptors have been identified and partially characterized, mapping antiviral blocks at multiple stages in virus entry [117]. Only recently has the first antiviral factor been identified that appears to block nuclear import of viral genomes. This factor referred to as myxovirus resistance protein 2 (MxB) is an interferon-inducible dynamin-like GTPase that inhibits HIV-1 infection [118,119,120,121]. Traditionally, MxB was thought to serve cellular functions, such as regulating cell cycle progression and nucleo-cytoplasmic trafficking [122,123]. Its N-terminal domain contains an NLS-like motif (residues 1–25) that causes the protein to preferentially localize to the nuclear envelope. Such nuclear targeting is strictly required for HIV-1 antiviral activity [120,124]. At the nuclear rim, MxB restricts HIV-1 by a poorly understood mechanism that occurs downstream of reverse transcription and involves the viral capsid. In the current working model, MxB acts as an HIV-1 capsid pattern sensor that associates with incoming viral capsids as an extended antiparallel dimer [125,126,127,128]. Binding and restriction are dictated solely by the N-terminal domain of MxB (91 residues) and GTPase activity is not required [119,120,129]. Cellular factors appear to somehow play a supporting role in MxB antiviral action. These include CypA, CPSF6, Nup153 and Nup358, which are known or suspected to affect the pathway used by HIV-1 DNA to enter the nucleus [130,131,132]. Conceivably, MxB may alter binding of these host factors to the viral capsid so as to interfere with proper uncoating and translocation at NPCs (Figure 2, lower right). Whether this is related to the observation that the preintegration viral complexes are reported to be positive for capsid antigens remains to be tested in the future [57]. Future work may also reveal how MxB impacts nuclear transport function and help define the role of innate immunity during viral nuclear entry.

## 5. Impact of Misdelivered Genomes on Viral Infections

As outlined above, problems encountered during nuclear entry may result in misdelivery, leading to accumulation of capsid-free unwound nucleic acids in the cytosol. In the following section, we consider the possible ways that both viruses and cells respond to such a situation and look at how misdelivered genomes may impact the course of viral infection. 

### 5.1. Cytosolic Sensing of Misdelivered Viral Genomes

The appearance of microbes and viruses has evolved an impressive arsenal of sensors and restriction factors that are strategically pieced together as a unified defense system to provide cell-wide protection against pathogens [9,111,133,134]. This host defense forced the pathogens to evolve countermeasures that come at a certain fitness cost. Viruses that misdeliver their DNA or RNA genome to the cytosol are subject to cytosolic nucleic acid sensors [135]. For instance, RNA sensors, such as melanoma differentiation factor 5 (MDA5) and retinoic acid-inducible gene I (RIG-I) discriminate self-RNA from foreign RNA by binding specific RNA structures, such as internal duplex regions of long dsRNA or uncapped 5′ PPP extremities, which are only found upon viral invasion. Likewise, cyclic GMP-AMP synthase (cGAS), interferon gamma-inducible protein 16 (IFI16), DNA-dependent activator of interferon-regulatory networks (DAI), DNA-dependent protein kinase and several other cytosolic receptors detect vDNA (for detailed reviews, see [136,137,138]). These different receptors would likely recognize unraveled misdelivered genomes and induce expression of proinflammatory cytokines and interferons to rally the cell’s defenses. This results in upregulation of hundreds of interferon-stimulated genes, including antiviral restriction factors and sensors themselves, as well as proteins important in regulating the immune response. At this point the cell stands a good chance at preventing viral infection, unless the virus antagonizes this antiviral response. 

### 5.2. Viral Measures against Misdelivery

Viruses can escape or block host innate detection or use it to their advantage [139,140]. This could be critical in the event of misdelivery at the NPC. Viral strategies that may be important for managing misdelivered genomes can be divided into at least three categories based on mode of action including: (1) masking nucleic acid structures recognized by the cell, (2) digesting nucleic acids trapped in the cytosol, and (3) targeting nucleic acid sensors and their downstream signaling molecules for deactivation or degradation. 

In terms of masking nucleic acids, viruses may avoid recognition by nucleic acid-sensing receptors through modifying their genomes to remove immune-stimulatory motifs, perhaps utilizing virus-encoded endonucleases/phosphatases [141], cytosine to thymine conversion [142], evolving under selective pressure [143], or by cloaking genomes with accessory proteins. Examples of cloaking candidates include the HAdV core protein VII and terminal protein, which stay associated with the viral genome during cell entry and appear to be important for protection [13,144].

Another possible way for viruses to handle misdelivery is to digest mislocalized genomes that would otherwise activate interferon expression. This can be accomplished by hijacking cellular factors, as HIV-1 does with the cytoplasmic exonuclease TREX1 [145], or possibly via manipulating an autophagic response [146,147]. 

Notably, the ability to utilize host proteins and pathways instead of relying on a limited copy number of viral factors to address misdelivery at the nuclear import stage is likely a critical determinant of infection. At this stage, viruses are only beginning to enter the nucleus and so very few, if any, viral proteins have been synthesized that can serve to antagonize innate or intrinsic factors in this early time of marked vulnerability. The window of vulnerability closes, when imported genomes trigger production of viral components that robustly target nucleic acid sensing pathways [148]. For instance, the multifunctional non-structural protein (NS1) of IAVs binds to tripartite motif 25 (TRIM25) and inhibits TRIM25-dependent RIG-I ubiquitination and downstream RIG-I signaling [149,150,151]. Interestingly, in addition to the described evasion strategies, at least one virus, HIV-1 also triggers collateral damage. Here, IFI16 detection of cytosolic vDNA induces a highly inflammatory form of programmed cell death referred to as pyroptosis, which causes dying CD4 T cells to release inflammatory signals that attract more T cells to die [152,153]. While viruses appear very capable of counterbalancing the consequences of misdelivery, the spatiotemporal aspects of cytosolic viral detection remain mysterious and for now we can only speculate about how this shapes the outcome of infection. 

## 6. Future Directions

Unveiling the precise mechanisms by which viruses import nucleic acids through NPCs is a long-standing goal for cell biologists, virologists and gene therapists alike. The cell biology bears conceptual interest due to the large size, hydrophilic nature and the tractability of the cargo. From a virology standpoint, gaining clues about how viruses manipulate cell-machinery to replicate and spread is critical in moving toward the goal of defining potential antiviral strategies. For gene therapy, this research informs new approaches for targeted delivery of exogenous genes into the nucleus of recipient cells. 

Recent novel labeling procedures combined with state-of-the-art super-resolution imaging have allowed a new direct view of viral nuclear import. Surprisingly, it was discovered that a significant fraction of incoming viruses fail to import their genomes into the nucleus upon attaching to NPCs [53]. This is an important finding that may have far-reaching implications for understanding virus-induced pathogenesis and for engineering virus-based gene delivery systems. 

Future work will aim to fill in structural data showing how viruses attach at NPCs and to define what types of structural rearrangements may ensue. The major challenge here is sample preparation, which necessitates capturing properly uncoated virus particles attached to the surface of NPCs. This will require extensive efforts in protocol development for infecting cells, isolating samples, and preserving them for experimentation. One promising avenue is cryo-electron tomography, which requires the additional sample preparation problem of maintaining the integrity of the sample while freezing in thin layers of ice (<1 µm thick) for imaging. Alternatively viral capsid-NPC sub-complexes can be purified and investigated in a divide-and-conquer strategy using traditional structural approaches such as X-ray crystallography, NMR spectroscopy, and/or single-particle cryo-electron microscopy. These experiments have the further limitation of size and flexibility, which may hinder determination of a high-resolution structure. 

In addition to structural studies, we will need to watch viral nucleic acids as they undergo transport through or misdelivery at NPCs in live-cells to quantify the kinetics and players involved in these processes. Live cell experiments are becoming increasingly possible with the availability of brighter fluorescent probes and new quantitative imaging methods that have high spatial and temporal resolution. Along with other tools such as ectopic expression and gene silencing, structural and live cell imaging experiments will serve to increase our understanding of virus misdelivery at nuclear pores and may reveal how cells guard against foreign DNA at the level of nuclear import. 
